# A Study of the Lipidomic Profiles of the CAL-27 and HOK Cell Lines Using EMS Spectra

**DOI:** 10.3389/fonc.2021.771337

**Published:** 2021-12-22

**Authors:** Xue-ying Wang, Ting Zhang, Wei-qun Guan, Hua-zhu Li, Ling Lin

**Affiliations:** ^1^ Department of Stomatology, Fujian Medical University Union Hospital, Fuzhou, China; ^2^ General Dentistry, School and Hospital of Stomatology, Fujian Medical University, Fuzhou, China; ^3^ Institutes of Biomedical Sciences of Shanghai Medical School, Fudan University, Shanghai, China

**Keywords:** lipidomic profile, CAL-27, HOK, cell lines, human tongue cancer

## Abstract

**Objective:**

The aim of this study was to explore the lipidomic profiles of the CAL-27 human tongue cancer cell line and the human oral keratinocyte (HOK) cell line.

**Methods:**

The lipidomic differences between the CAL-27 and the HOK cell lines were investigated using non-targeted high-performance liquid chromatography–mass spectrometry lipidomic analysis. The resulting data were then further mined *via* bioinformatics analysis technology and metabolic pathway analysis was conducted in order to map the most affected metabolites and pathways in the two cell lines.

**Results:**

A total of 711 lipids were identified, including 403 glycerophospholipids (GPs), 147 glycerolipids, and 161 sphingolipids. Comparison of the enhanced MS (EMS) spectra of the two cell lines in positive and negative ionization modes showed the lipid compositions of HOK and CAL-27 cells to be similar. The expressions of most GP species in CAL-27 cells showed an increasing trend as compared with HOK, whereas a significant increase in phosphatidylcholine was observed (*p* < 0.05). Significant differences in the lipid composition between CAL-27 and HOK cells were shown as a heatmap. Through principal component analysis and orthogonal partial least squares discriminant analysis, noticeably clear separation trends and satisfactory clustering trends between groups of HOK and CAL-27 cells were identified. The numbers of specific lipid metabolites that could distinguish CAL-27 from HOK in positive and negative modes were 100 and 248, respectively. GP metabolism was the most significantly altered lipid metabolic pathway, with 4 metabolites differentially expressed in 39 hit products.

**Conclusion:**

This study demonstrated the potential of using untargeted mass spectra and bioinformatics analysis to describe the lipid profiles of HOK and CAL-27 cells.

## Introduction

Oral cancer is one of the most common human cancers and is associated with an overall 5-year survival rate of less than 50% (1). Oral squamous cell carcinoma (OSCC) develops from oral potential malignant disorders (OPMDs) in a stepwise model and has the highest morbidity of all oral cancer types. Since it is usually asymptomatic, patients are often diagnosed at an advanced stage. Screening high-risk populations to detect OSCC and OPMDs at an early stage is therefore sensible. In recent years, considerable research has been undertaken to understand the genetic, proteinic, and lipidomic basis of OSCC. A number of specially expressed genes and proteins have been proposed as biomarkers for use in clinical diagnosis (2–4). However, these approaches have usually shown insufficient diagnostic sensitivity and specificity.

Lipids, one of the essential cellular components, play important roles in cell function, acting as a biological barrier and being involved in signal conduction, substance transportation, and energy storage (5). The composition of lipids depends on the different types of cells and tissues. Lipid composition may change in different physical conditions. Variations in genetic status and protein expression can also result in changes in particular lipids (5–12).

Lipids are the most complicated molecules in terms of chemical structure. The classification of a lipid depends on both the headgroup and the type of linkage between the headgroup and the acyl chains (13). Variations in the acyl chain and the headgroup regulate an extremely large number of individual lipid molecular species. There are currently more than 1.5 million lipids found in the lipid database, while only hundreds of species could be mapped using existing technology (14).

Mass spectrometry (MS) with high-performance liquid chromatography (HPLC) is used for lipid profiling (15), as well as for its reducing ion suppression effects (16). Non-targeted HPLC-MS lipidomic analysis enables the detection of “all” ion features in biological samples. Bioinformatics analysis aids in the simplification of the data and identifies the significantly affected lipids at the start of the research, making the work more efficient. Therefore, this study investigated the lipidomic profiles of the CAL-27 human tongue cancer cell line and the human oral keratinocyte (HOK) cell line in an attempt to explore whether the lipid markers identified by untargeted MS and bioinformatics analysis could be used in the early detection of OSCC.

## Materials and Methods

### Materials

GC grade dichloromethane (DCM), ammonium acetate, and ammonium hydroxide and 2-propanol (IPA) were supplied by Fisher Scientific (Waltham, MA, USA). LC-MS grade methanol (MeOH) and methyl *tert*-butyl ether (MTBE) were supplied by CNW Technologies (Stuart, FL, USA). Lipid standards including d7-PE, d7-LPC, and d7-TG were supplied by Avanti Polar Lipids (Alabaster, AL, USA).

The CAL-27 cell line was provided by Professor Youguang Lu of the Stomatology School, Fujian Medical University. The HOK cell line was provided by Ruijin Hospital, Shanghai Jiao Tong University School of Medicine.

### Cell Culture

The two cell lines were cultured in Dulbecco’s modified Eagle’s medium containing 10% fetal bovine serum (Gibco BRL, Indianapolis, IN, USA), 100 IU/ml penicillin G, and 100 mg/ml streptomycin sulfate (Hyclone, Logan, UT, USA). Cells were cultured at 37°C with 5% CO_2_ (SANYO MCO-17A, Japan SL Shel LAB), and the culture medium was replaced every 2 or 3 days.

### Cell Lipid Extraction

Each cell pellet (~5 × 10^6^ cells/sample) was mixed with 400 μl water, and the sample was then incubated in liquid nitrogen for 1 min, then thawed at room temperature. This freezing–thawing cycle was repeated three times in total. Then, 960 μl of the extraction solvent (MTBE/MeOH = 5:1, *v*/*v*), including 10 μl 10 ppm d7-PE (15:0/18:1), 2 μl 10 ppm d7-LPC (18:1), and 4 μl 10 ppm d7-TG (15:0/18:1/15:0), was added. The sample was vortexed for 30 s, followed by 10 min of sonication. The solution was then centrifuged at 3,000 rpm for 15 min. The upper organic layer (i.e., the MTBE layer) was collected into a new Eppendorf tube. Then, 500 μl of MTBE was added to the left aqueous layer for further extraction. The solution was vortexed, sonicated, and centrifuged three times, as previously described. Finally, the pooled organic layer was evaporated. The dry extract was reconstituted using 100 μl of DCM/MeOH (1:1, *v*/*v*) prior to liquid chromatography tandem MS (LC-MS/MS) analysis. 60μl of the solvent was carefully transferred into a 2-ml tube. Of each sample, 10 μl was mixed to form pooled quality control (QC) samples, then 60 μl was taken for further analysis.

### Chromatography and Mass Spectrometry

LC-MS/MS analysis was performed using an HPLC system (1290 series, Agilent Technologies, Santa Clara, CA, USA) coupled with quadrupole time-of-flight mass spectrometry (TripleTOF 6600; AB Sciex, Redwood City, CA, USA). Lipid extract separations were performed on a Phenomenex Kinetex C18 column (1.7 μm particle size, 100 mm in length × 2.1 mm i.d.) with a column temperature of 55°C. The injection volume was 3 μl in negative mode and was 0.5 μl in positive mode. Mobile phase A was 10 mM ammonium formate in H_2_O/ACN (4:6, *v*/*v*) and mobile phase B was 10 mM ammonium formate in IPA/ACN (9:1, *v*/*v*). The two mobile phases were used for electrospray ionization in positive and negative modes. The linear gradient eluted from 40% to 100% B (0–12 min), 100% B (12–13.5 min), and from 100% to 40% B (13.5–13.7 min), and then equilibrated at 40% B until 18 min. The flow rate was set at 0.3 ml/min.

High-resolution MS and MS/MS data were acquired using TripleTOF 6600 MS based on an information-independent acquisition function under the control of the software Analyst TF (version 1.7.1, AB Sciex). The MS ion intensity and counts larger than 100 were further dissociated for MS/MS at every acquisition cycle. The dissociation energy was set to 35 eV for the generation of the tandem mass spectra (MS2), and the speed of the MS2 scan was set to 500 counts every 50 ms. The parameters of electrospray ionization were set as follows: ion source gas 1, 60 Pa; ion source gas 2, 60 Pa; curtain gas, 30 Pa; temperature, 550°C; ion spray voltage floating, 5,500 V in positive mode and −4,500 V in negative mode.

The raw data files (wiff format) were converted into files in mzXML format using the msconvert program from ProteoWizard, version 3.0.6150. Then, the mzXML files were loaded into LipidAnalyzer for data processing, which was developed using R for automatic data analysis. The isotope signals and repeated signals containing potassium ions, sodium ions, and ammonium ions were removed during the analysis. Peak detection was first applied to the MS1 data. The centWave algorithm in XCMS was used for peak detection. The cutoff for matching scores was set at 0.6 and the minifrac was set at 0.5. With the MS/MS spectrum, lipid identification was achieved through a spectral match using an in-house MS/MS spectral library. The preprocessing results generated a data matrix that consisted of the retention time, the mass-to-charge ratio (*m*/*z*), and the peak intensity. The absolute quantitation of lipids can be achieved using the peak area, stable isotope-labeled internal standard (SIL-IS), and response factor (RF) information.

### Statistical Analyses

Data were expressed as the mean ± standard deviation. After data processing, univariate and multivariate statistical analyses were performed to screen the significant differentially expressed features. Those features were then confirmed by searching LIPID MAPS and the HMDB database and matching standards and the targeted data-dependent acquisition spectra. SIMCA-P 14.1 (Umetrics, Umca, Sweden) was used for multivariable analysis, including principal component analysis (PCA) with mean-centered scaling and orthogonal partial least squares discriminant analysis (OPLS-DA) with unit variance scaling. As well as generally interpreting the clustering trend for the multidimensional data, PCA was carried out to reduce the dimensionality of the dataset. OPLS-DA was then applied to detect global lipid differences among CAL-27 and HOK cells; at the same time, the corresponding variable importance in projection (VIP) values were calculated in the OPLS-DA model. The criteria for selected potential metabolic biomarkers were set as follows: a VIP value greater than 1 and a *p*-value less than 0.05 using Student’s *t*-test. In addition, by conducting a further search of commercial databases, such as the Kyoto Encyclopedia of Genes and Genomes database (KEGG; http://www.genome.jp/kegg/), large quantities of differential metabolites were cross-mapped to the pathways.

## Results

### Positive and Negative Electrospray Ionization MS/MS

**Figure 1:**
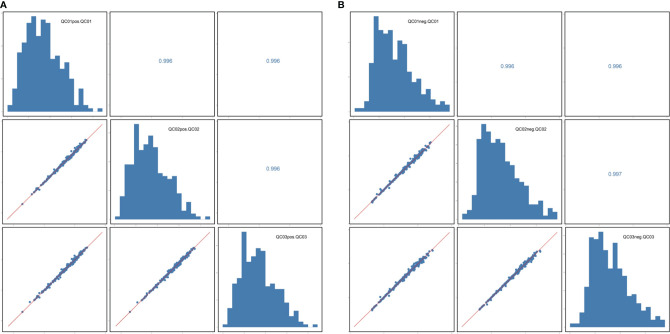
Correlation analysis of quality control samples. **(A)** Positive mode. **(B)** Negative mode.

**Figure 2:**
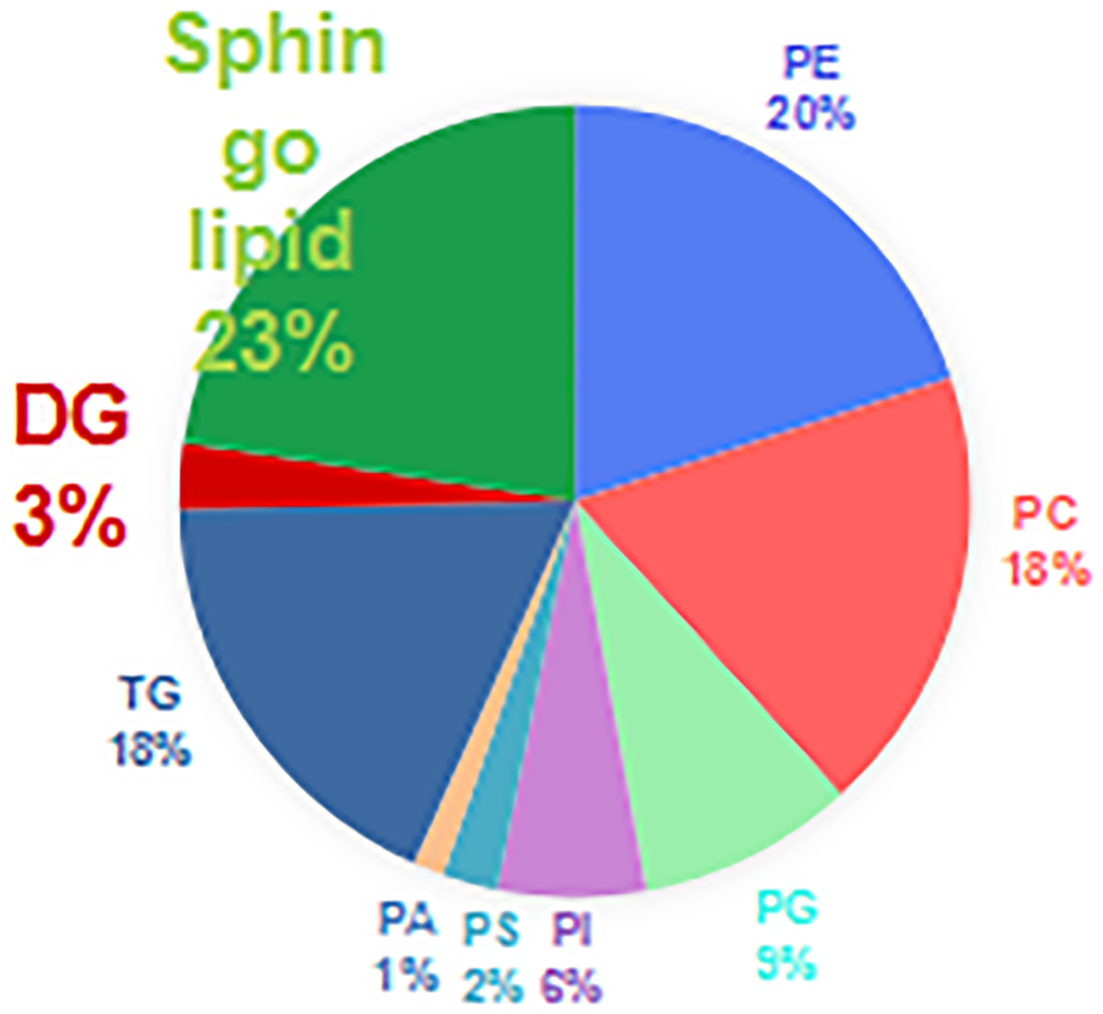
Lipid compositions of the CAL-27 and human oral keratinocyte cell lines.

The correlation coefficient of the QC samples was close to 1, indicating that the QC samples were tightly clustered (**Figures 1A, B**). A total of 711 lipids were identified, including 403 glycerophospholipids (GPs), 147 glycerolipids, and 161 sphingolipids (SPs) (**Figure 2**). Comparing the enhanced MS (EMS) spectra of the two cell lines in positive/negative ionization modes, it was found that the lipid compositions of HOK and CAL-27 cells were similar.

### Relative Quantitative Analysis

**Figure 3:**
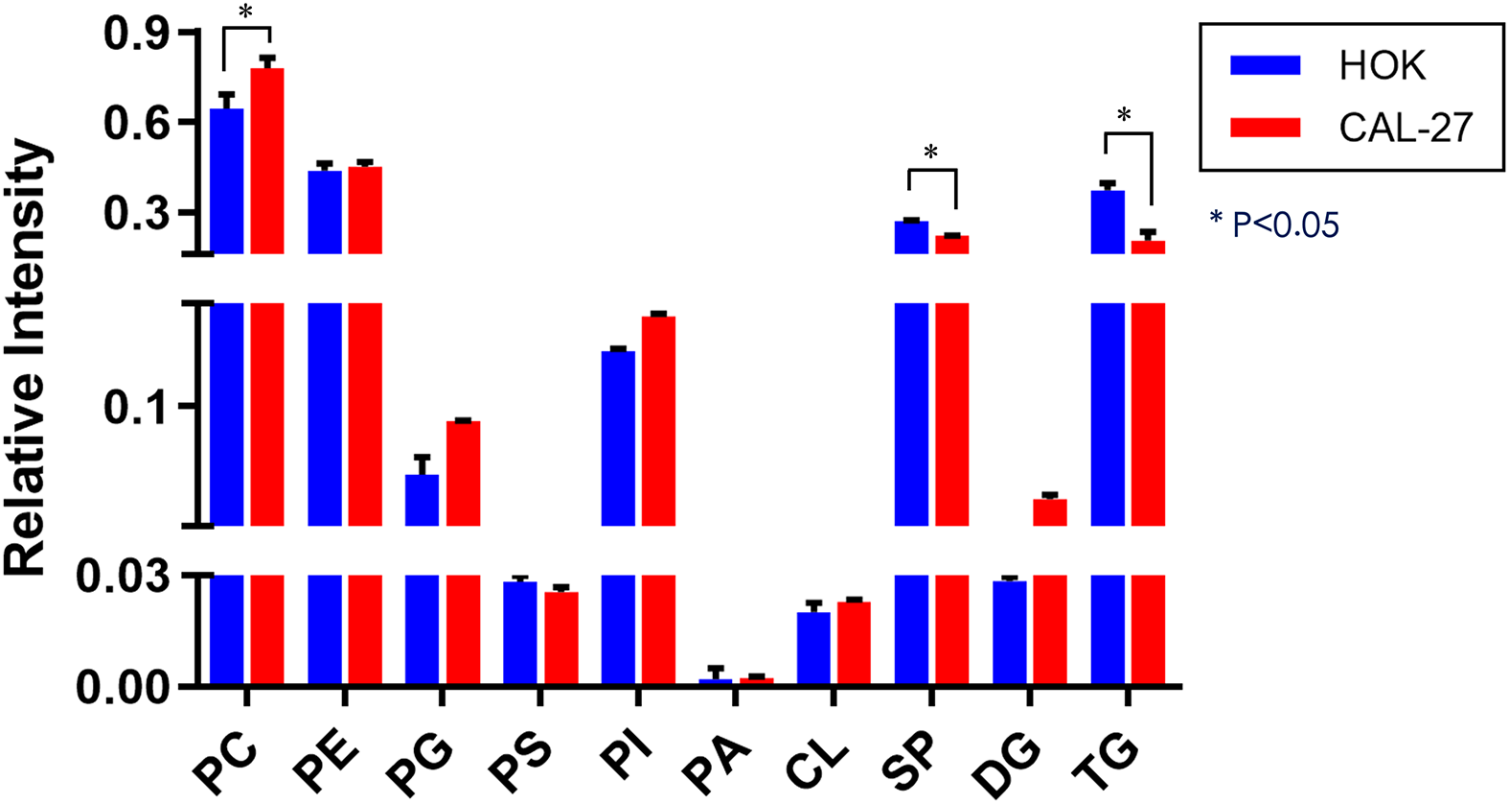
Differential lipidomic metabolites in the CAL-27 and human oral keratinocyte cell lines.

The expressions of diglyceride (DG) and most of the GP species [phosphatidylcholine (PC), phosphatidylglycerol (PG), and phosphatidylinositol (PI)] in CAL-27 cells showed an increasing trend as compared with those of HOK cells, whereas a significant increase in PC was observed (*p* < 0.05; **Figure 3**). The expressions of phosphatidylserine (PS), SP, and triglyceride (TG) were downregulated in the CAL-27 cell line, and among them, the expressions of SP and TG were both significantly decreased (*p < 0.05; **Figure 3**).

### Multivariate Data Analysis

**Figure 4:**
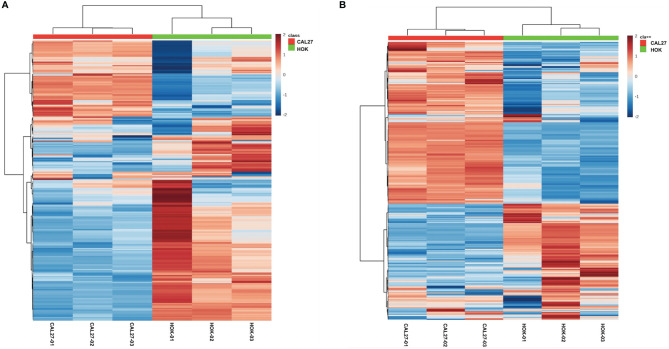
Differentially expressed lipid metabolites in the CAL-27 and human oral keratinocyte cell lines. **(A)** Positive mode. **(B)** Negative mode.

Significant differences between CAL-27 and HOK cells are shown as a heatmap in **Figures 4A, B**, ranked by variance analysis. Each row represents an individual lipid, and each column represents an individual sample. The lipid compositions of the two cell lines were similar. However, they showed quite different expression trends in each lipid, and this may have resulted in the different biological properties seen between the two cell lines.

### PCA

**Figure 5:**
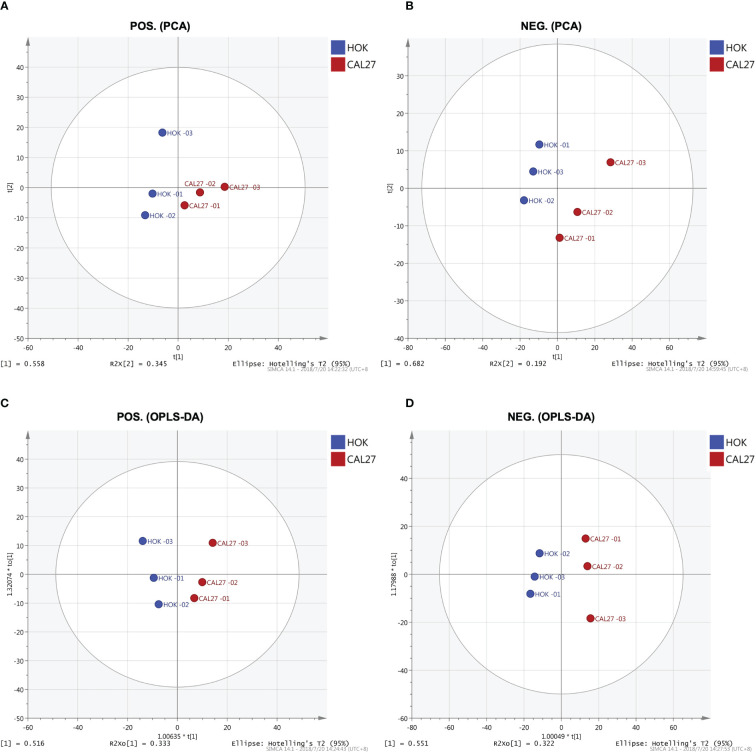
Lipid profiles of the CAL-27 and human oral keratinocyte cell lines. **(A)** Principal component analysis (PCA) in positive mode. **(B)** PCA in negative mode. **(C)** Orthogonal partial least squares discriminant analysis (OPLS-DA) in positive mode. **(D)** OPLS-DA in negative mode.

Through PCA, clear separation trends between groups of HOK and CAL-27 cells were identified (**Figures 5A, B**). Subsequently, a supervised method, OPLS-DA, was applied in the data analysis. As can be seen in **Figures 5C, D**, satisfactory clustering trends among HOK and CAL-27 cells were observed in the scores plot.

### Lipid Metabolite Features

**Figure 6:**
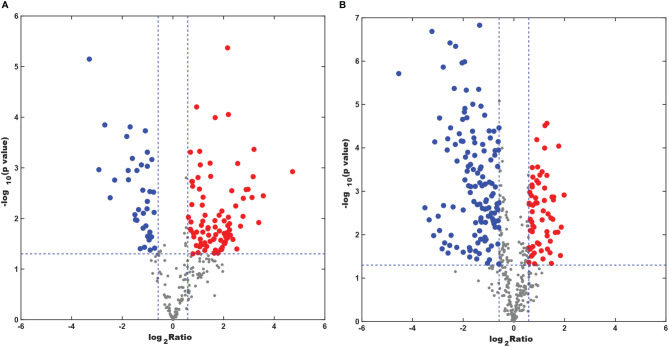
Differentially expressed lipid metabolites in the CAL-27 and human oral keratinocyte cell lines. **(A)** Positive mode. **(B)** Negative mode.

The numbers of specific lipid metabolites that could distinguish CAL-27 from HOK in positive and negative modes were 100 and 248, respectively (**Supplementary Table 1, 2**). In the volcano plots, each spot represents an individual lipid. The red spots represent the lipids undergoing a fold change increase of more than 1.5, the blue spots indicate the lipids that declined by more than 1.5-fold, and the gray spots represent the lipids that did not demonstrate significant changes (*p* < 0.05). The volcano plots above showed visually that there were some lipid metabolites that had the potential to discriminate between the two cell lines (**Figures 6A, B**). A combination of differentially expressed lipid metabolites would be more sensitive and specific for biomarker selection.

### Disturbed Metabolic Pathway

Topological analysis of the differentially expressed lipid metabolites was conducted using the MetaboAnalyst platform. Then, based on the KEGG database, pathway analysis was performed. The detailed results are listed in **Tables 1**, **2**. SP metabolism was disturbed significantly in positive mode (impact > 0.1). The only differentially expressed metabolites among the 25 mapped lipids was ceramide (d18:1/20:1;cpd:C00195), which was upregulated. On the other hand, in the negative mode, GP metabolism emerged at the top of the significantly altered lipid metabolic pathways. There were four metabolites differentially expressed in 39 hit products. Phosphatidylethanolamine (PE) (cpd: C00350) was upregulated, while PC (cpd: C00157), PA (16:0/16:0; cpd: C00416), and PS (16:0/16:0; cpd: C02737) showed a decrease. According to the criteria, and using a pathway impact larger than 0.1 and a *p*-value less than 0.05, GP metabolism in negative mode showed significant changes.

**Table 1:** Relative quantifications of 100 differentially expressed lipids in the CAL-27 cell line *versus* the human oral keratinocyte cell line in positive mode.

Pathway	Total	Hits	Raw *p*-value	−ln(*p*)	FDR	Impact
Sphingolipid metabolism	25	1	0.050906	2.9778	1	0.29423
Glycerolipid metabolism	32	1	0.064781	2.7367	1	0.05145
Glycerophospholipid metabolism	39	1	0.078494	2.5447	1	0.00317

FDR, false discovery rate.

**Table 2:** Relative quantifications of 248 differentially expressed lipids in the CAL-27 cell line *versus* the human oral keratinocyte cell line in negative mode.

Pathway	Total	Hits	Raw *p*-value	−ln(*p*)	FDR	Impact
Glycerophospholipid metabolism	39	4	2.9135E−07	15.049	0.000023308	0.3663
GPI anchor biosynthesis	14	1	0.028769	3.5485	0.82129	0.0439
Linoleic acid metabolism	15	1	0.030798	3.4803	0.82129	0
alpha-Linolenic acid metabolism	29	1	0.058855	2.8327	1	0
Glycerolipid metabolism	32	1	0.064781	2.7367	1	0.01247
Glycine, serine, and threonine metabolism	48	1	0.095887	2.3446	1	0
Arachidonic acid metabolism	62	1	0.12242	2.1003	1	0

FDR, false discovery rate; GPI, glycosylphosphatidylinositol.

## Discussion

In this study, the correlation coefficient of the QC samples was close to 1, indicating that they were tightly clustered, and this demonstrated the stability and reliability of the lipid profiling platform in this study. Comparison of the EMS spectra of the two cell lines in positive and negative ionization modes showed the lipid compositions of HOK and CAL-27 cells to be similar. The overall similarity of the lipid profiles between cancerous and normal cell lines revealed the similar basic composition of the lipid membranes in both cell types.

To obtain an overview of the association between the major lipid composition of the two different cell lines, we performed relative quantitative analysis. The expressions of DG and most of the GP species (PC, PG, and PI) in CAL-27 cells had a growing trend compared with those in HOK cells, whereas a significant increase in PC was observed. The expressions of PS, SP, and TG were downregulated in the CAL-27 cell line, and among them, the expressions of SP and TG showed a significant decrease. PC, as the main plasma membrane phospholipid, accounts for approximately 50% of the total cellular phospholipids and is the most abundant phospholipid in mammalian membranes. The robust increase of PC in CAL-27 cells may predicate the high proliferation of tumor cells.

Research by Wang et al. indicated that, in the plasma of OSCC patients, all GPs were decreased compared with that in healthy controls, especially PC and phosphoethanolamine plasminogen. In contrast, the SPs were increased, and among them, there were 12 lipids associated with pathological staging that could be used in the early diagnosis of OSCC (17). Uchiyama et al. identified that visualizing PC (16:0/16:1) and PC (18:1/20:4) could identify the border between the cancer and stromal regions of OSCC using MS imaging (18). The imaging of lipidome components distinguished oral cancer from normal epithelium in tissue samples using matrix-assisted laser desorption/ionization (MALDI) MS imaging, indicating that the lipidome components in cancer and normal samples of OSCC were different (19). It appears that the results in this study were different from those of other lipidomics research studies using other OSCC samples. The differences in the lipid compositions may in part have resulted from the use of different sample types. Apart from cancer, tissue or blood samples could be affected by many factors, such as inflammation and the immune response (20). Some studies identified that total plasma cholesterol and high-density and low-density lipoproteins decreased significantly in OSCC patients compared with those in normal subjects, while there was no significant difference in the plasma TG (21–23).

In order to further mine the data, multivariate data analysis (MDA) methods were used for the visualization of the differences and similarities between normal and tumor cell lines. As with the heatmap, the PCA and OPLS-DA, volcano plots, the MDA methods showed that the lipid compositions of the two cell lines were similar, although they showed different expression trends in each lipid, and this may have resulted in the different biological properties between the two cell lines. Thus, it showed the potential to differentiate between the two cell lines by lipidomic analysis. Through PCA and OPLS-DA, clear separation trends and satisfactory clustering trends between groups of HOK and CAL-27 cells were identified, indicating the potential for using lipidomic analysis to distinguish between two different cells. From the volcano plots above, it was observed that the numbers of specific lipid metabolites that could distinguish CAL-27 from HOK in positive and negative modes were 100 and 248, respectively.

According to the criteria, using a pathway impact larger than 0.1 and a *p*-value less than 0.05, GP metabolism in negative mode showed significant changes. It is necessary to further investigate the changes in GP metabolism in the lipid metabolism profile. As the largest class of phospholipids in organisms, GPs and the metabolic pathways have always been the focus of lipidomic histology. There is a complex transformation relationship between them. PC and PE could be investigated using the Kennedy pathway or the cytidine diphosphate diacylglycerol (CDP-DAG) pathway. Moreover, PC, PE, and PS could transform into each other under certain conditions (20–23). Therefore, MS analysis coupled with bioinformatics analysis will help in identifying the targets and key pathways for further research. The efficiency would be higher when combining clinical information.

There are some differences between the results from the untargeted lipidomic analysis and those from the bioinformatics analysis. We annotated these for the different data processing and cutoff value standards. On the other hand, untargeted MS analysis for lipidomic composition in the two cell lines may have some influence on the accuracy of the results. Consequently, it is suggested that a targeted lipid analysis for the significantly affected lipid molecular species, which have been mapped in differentially expressed metabolic pathways, be used. A large number of studies in the past 2 years have suggested that tumor cells have undergone metabolic remodeling, not only *via* the Warburg effect of glucose metabolism in tumor cells, but more and more research groups have reported that fat metabolism is abnormally upregulated in tumor cells, while lipidomic profiling of oral cancer cells has not yet been elucidated. This study has shown that oral cancer cells with different metastatic potentials can be found to have characteristic fatty acid chain lengths and different lipid compositions, and this has broadened the understanding of oral cancer.

## Conclusion

Lipid metabolism in cancer cells or cancerous samples remains largely unclear. This study has demonstrated the potential to describe the lipid profiles of HOK and CAL-27 cells using untargeted MS and bioinformatics analysis. The primary purpose of this work was to construct a reliable and sensitive MS platform, not to detect a specific lipid or metabolic pathway. The methodology used in this study to profile the lipid metabolites of the cell lines is not limited to biomarker selection, but also offers the potential to describe OSCC by integrating histological or clinical features.

## Data Availability Statement

The original contributions presented in the study are included in the article/**Supplementary Material**. Further inquiries can be directed to the corresponding author.

## Ethics Statement

This study was conducted in accordance with the Declaration of Helsinki. This study was conducted with approval from the Ethics Committee of Fujian Medical University Union Hospital University. Written informed consent was obtained from all participants.

## Author Contributions

X-YW and TZ conceptualized and designed the study, drafted the initial manuscript, and reviewed and revised the manuscript. H-ZL and LL designed the data collection instruments, collected data, carried out the initial analyses, and reviewed and revised the manuscript. W-QG coordinated and supervised data collection and critically reviewed the manuscript for important intellectual content. All authors approved the final manuscript as submitted and agree to be accountable for all aspects of the work. All authors have contributed significantly to the manuscript and declare that the work is original and has not been submitted or published elsewhere.

## Conflict of Interest

The authors declare that the research was conducted in the absence of any commercial or financial relationships that could be construed as a potential conflict of interest.

## Publisher’s Note

All claims expressed in this article are solely those of the authors and do not necessarily represent those of their affiliated organizations, or those of the publisher, the editors and the reviewers. Any product that may be evaluated in this article, or claim that may be made by its manufacturer, is not guaranteed or endorsed by the publisher.

## Supplementary Material

Supplementary Table 1 Relative quantifications of 100 differential expressed lipids in CAL-27 cell line versus HOK cell line in positive mode.

Supplementary Table 2 Relative quantifications of 248 differential expressed lipids in CAL-27 cell line versus HOK cell line in negative mode.

Supplementary Table 3 the original mass spectrometry analysis data in cationic mode.

Supplementary Table 4 the original mass spectrometry analysis data in anion mode.
